# Investigation of the Relationship Between *Fok1* and *Col1A1* Gene Polymorphisms and Development of Treatment-Related Bone Complications in Children with Acute Lymphoblastic Leukemia

**DOI:** 10.4274/tjh.galenos.2018.2018.0221

**Published:** 2019-02-07

**Authors:** Melek Erdem, Özlem Tüfekçi, Sefa Kızıldağ, Şebnem Yılmaz, Deniz Kızmazoğlu, Berna Eroğlu Filibeli, Hale Ören

**Affiliations:** 1 Dokuz Eylül University Faculty of Medicine, Department of Pediatric Hematology, İzmir, Turkey; 2 Dokuz Eylül University Faculty of Medicine, Department of Medical Biology, İzmir, Turkey; 3 Dokuz Eylül University Facullty of Medicine, Department of Pediatrics, İzmir, Turkey

**Keywords:** Acute lymphoblastic leukemia, Bone mineral density, Genetic polymorphism, Osteonecrosis, Osteoporosis

## Abstract

**Objective:**

In acute lymphoblastic leukemia (ALL), various clinical risk factors and genetic predispositions contribute to the development of bone complications during and after chemotherapy. In this study, we aimed to investigate whether vitamin D receptor (*VDR*) *Fok1* and collagen protein *Col1A1* Sp1-binding site gene polymorphisms, which are important in bone mineral and matrix formation, have effects on the development of bone abnormalities in childhood ALL survivors.

**Materials and Methods:**

Fifty children with ALL who were treated with the ALL Berlin-Frankfurt-Muenster-95 protocol between 1998 and 2008 and were followed for at least 7 years were enrolled. The control group consisted of 96 healthy children. *VDR Fok1* and *Col1A1* Sp1-binding site gene polymorphisms were analyzed by polymerase chain reaction and restriction fragment length polymorphism. Bone mineral density (BMD) and markers of bone metabolism were all noted. All patients who presented with pain in the joints were examined for bone pathologies while on chemotherapy or during long-term follow-up.

**Results:**

Low BMD (16%), osteoporosis (12%), and osteonecrosis (8%) were present in a total of 18 patients (36%). The frequency of osteonecrosis and total bone abnormalities was significantly higher in children aged ≥10 years (p=0.001). The risk of low BMD and osteonecrosis was higher in those with vitamin D deficiency. Only the *Col1A1* Sp1-binding site gene polymorphism showed a significant association in ALL patients with osteonecrosis.

**Conclusion:**

The development of therapy-induced bone mineral loss and osteonecrosis in children with ALL is frequent and the risk is especially higher in children aged ≥10 years and with vitamin D deficiency. The association between *Col1A1* Sp1-binding site gene polymorphisms and osteonecrosis has to be assessed in a larger group of ALL survivors.

## Abstract (tr)

**Amaç:**

Akut lenfoblastik lösemide (ALL), çeşitli klinik risk faktörleri ve genetik yatkınlıklar kemoterapi sırasında ve sonrasında kemik komplikasyonlarının gelişmesine katkıda bulunur. Bu çalışmada, kemik mineral ve matriks oluşumunda önemli olan D vitamini reseptörü (VDR) *Fok1* ve kollajen proteini *Col1A1* Sp1 bağlayıcı bölge gen polimorfizmlerinin, ALL’den sağ kalan çocuklarda kemik anormalliklerinin gelişimine etkilerinin olup olmadığını araştırmayı amaçladık.

**Gereç ve Yöntemler:**

1998-2008 yılları arasında ALL tanısı alıp, ALL Berlin-Frankfurt-Muenster-95 protokolü ile tedavi edilen ve en az 7 yıl takip edilen 50 çocuk çalışmaya dahil edildi. Kontrol grubu 96 sağlıklı çocuktan oluşuyordu. *VDR Fok1* ve *Col1A1* Sp1 bağlayıcı bölge gen polimorfizmleri polimeraz zincir reaksiyonu ve restriksiyon fragman uzunluğu polimorfizmi ile analiz edildi. Kemik mineral yoğunluğu (KMY) ve kemik metabolizması belirteçleri kaydedildi. Eklemlerde ağrı ile başvuran tüm hastalar kemoterapi veya uzun süreli takip sırasında kemik patolojileri açısından incelendi.

**Bulgular:**

Toplam 18 hastada (%36) düşük KMY (%16), osteoporoz (%12) ve osteonekroz (%8) mevcuttu. Osteonekroz sıklığı ve toplam kemik anormallikleri 10 yaş ve üstü çocuklarda anlamlı olarak yüksek bulundu (p=0,001). D vitamini eksikliği olanlarda düşük BMD ve osteonekroz riski daha yüksekti. Gen polimorfizmleri ile ilişki incelendiğinde; sadece *Col1A1* Sp1 bağlayıcı bölge gen polimorfizmi ile osteonekroz arasında anlamlı bir ilişki gösterilmiştir.

**Sonuç:**

Sonuç olarak, ALL’den sağ kalan çocuklarda tedaviye bağlı kemik mineral kaybı ve osteonekroz gelişimi sıktır ve bu risk özellikle 10 yaş ve üstü D vitamini eksikliği olan çocuklarda daha yüksektir. *Col1A1* Sp1 bağlanma bölgesi gen polimorfizmleri ve osteonekroz arasındaki ilişkiyi araştırmak için ALL’den sağ kalan bireyler üzerinde yapılacak daha geniş çaplı çalışmalara ihtiyaç vardır.

## Introduction

Cure rates for childhood acute lymphoblastic leukemia (ALL) have approached 90% with therapeutic advances over the last several decades and the number of survivors has dramatically increased over the last decades [1,2]. Many treatment-related long-term complications including impaired physical growth, neurocognitive dysfunction, cardiac abnormalities, secondary neoplasms, low bone mineral density (BMD), osteoporosis, and osteonecrosis have been reported [3,4,5]. Bone infiltration of leukemic cells, corticosteroids, methotrexate (MTX) and asparaginase exposure, poor nutrition, low vitamin D, poor muscle mass, and genetic predisposition contribute to the development or worsening of bone pathologies during or after therapy [3,4]. Multiple clinical risk factors including female sex, administration of dexamethasone, and age have also been identified to have roles in the development of osteoporosis and osteonecrosis [5,6,7,8,9,10]. 

Corticosteroids, which play a critical role in ALL therapy, directly affect bone and negatively impact the skeleton by altering the hormonal axis, intestinal calcium absorption, and renal excretion of calcium. Multiple candidate gene studies have indicated several polymorphisms in genes putatively related to the development of osteonecrosis, such as *SERPINE* 1, vitamin D receptor (*VDR*), and *CYP3A4* [11,12]. 

Vitamin D plays a major role in calcium, phosphorus, and bone metabolism and thus is an important variable in the assessment of bone health [13]. Vitamin D is an important factor that mediates its action in the body through *VDR*, which helps in calcium uptake or bone formation like calcium binding proteins and osteocalcin [14]. The *VDR* *Fok1* locus polymorphism is considered to be a potential regulator of bone and calcium metabolism. Some studies have suggested significant association of *Fok1* locus polymorphism with low BMD in girls, whereas others showed no such association [15,16,17,18]. 

The main component of bone mineral is calcium, and for bone matrix, it is collagen. Osteoporosis is mainly due to the loss of calcium and collagen degradation [19]. The *Col1A1* gene encodes the alpha-1 protein chain of type I collagen, the major protein of bone [20]. Some research has focused on the *Col1A1* Sp1-binding site polymorphism and *Col1A1* upstream regulatory region single nucleotide polymorphisms, mainly because they can regulate the expression of the *Col1A1* gene. These polymorphisms have been significantly associated with low BMD, osteoporosis, and increased fracture risk [19,20,21,22,23]. 

In this study, we aimed to investigate whether *VDR* *Fok1* and collagen protein *Col1A1* Sp1-binding site gene polymorphisms, which are important in bone mineral and matrix formation, have effects on the development of bone abnormalities in childhood ALL survivors.

## Materials and Methods

### Study Design and Patients

Fifty children with ALL who were diagnosed and treated with the ALL Berlin-Frankfurt-Muenster (BFM) 95 protocol [24] between 1998 and 2008 and were followed for at least 7 years after cessation of therapy were enrolled in this study. The control group consisted of 96 healthy children of similar age and sex. The children in the control group had no malignant tumors, chronic diseases, or musculoskeletal system symptoms and also had no evidence of vitamin D deficiency or hypocalcemia. 

In the ALL-BFM 95 protocol, patients were stratified according to age, initial white blood cell count, day 8 response to prednisone, immunophenotype, and molecular rearrangements such as t(9;22) and t(4;11) into standard-risk (SRG), medium-risk (MRG), and high-risk (HRG) groups. SRG and MRG therapy consisted of an 8-drug induction including prednisone, consolidation with a four-times higher dose of MTX, and an 8-drug reintensification including dexamethasone. HRG patients were treated with a 5-drug induction including prednisone, followed by six intensive multiagent blocks; reintensification was similar to that for the SRG and MRG patients. Maintenance therapy consisted of daily 6-MP and weekly MTX and was continued until 2 years after initial diagnosis. Patients were evaluated for age, sex, risk group, relapse, hemopoietic stem cell transplantation (HSCT), bone metabolism markers (serum calcium, phosphorus, alkaline phosphatase, parathyroid hormone, and 25-hydroxyvitamin D levels), bone changes (clinical and radiographic findings), and *Fok1* and *Col1A1* Sp1-binding site gene polymorphisms. Vitamin D levels below 20 ng/mL were considered as deficiency.

In this study, low BMD, osteoporosis, and osteonecrosis were investigated as bone complications. BMD and markers of bone metabolism were all screened routinely before initiation of maintenance treatment and were studied whenever clinically needed. All patients who presented with pain in the joints were examined for bone pathologies while on chemotherapy or during long-term follow-up. All of the data were noted and collected from hospital records. Diagnosis of osteonecrosis had been made based on symptoms, clinical exam findings, and radiographic studies, including plain radiographs and magnetic resonance imaging (MRI). Diagnosis of low BMD and osteoporosis was made based on clinical findings, dual-energy X-ray absorptiometry (DEXA), and radiographic studies [25]. The BMDs were measured in g/cm^2^ and converted to Z-scores, which represent deviation from age-matched and sex-matched normative BMDs. Mean values and standard deviations (SDs) were calculated for each patient. We deﬁned osteoporosis as a BMD ≤2 SD below the mean (Z ≤ -2) and low BMD as a BMD that is abnormal but not >2 SD below the mean (-2> Z ≤0) [36]. The term “osteopenia” was not used since “low BMD” is the preferred term for pediatric DEXA reports [26]. In our hospital DEXA results were assessed by measuring mineral density in the L1-L4 vertebra corpus; therefore, the Z-scores in the definition of osteoporosis and low BMD were obtained by this regional analysis. The proximal femur or hip region was included according to clinical and radiologic findings.

Informed consent was obtained from the parents of all patients. This study was approved by the Ethics Committee of the Dokuz Eylül University Faculty of Medicine and the work described was carried out in accordance with the Declaration of Helsinki for experiments involving humans.

### Genotyping

Peripheral blood samples from all individuals were collected into sterile tubes containing 0.1 M EDTA and stored at -20 °C. Genomic DNA was extracted using the NucleoSpin Blood Extraction Kit (Macherey-Nagel, Duren, Germany). The genotypes were detected by polymerase chain reaction-restriction fragment length polymorphism (PCR-RFLP) analysis. The polymorphic locus was amplified using 5¢-AGCTGGCCCTGGCACTGACTCTGCTCT-3¢ as the forward and 5¢-ATGGAAACACCTTGCTTCTTCTCCCTC-3¢ as the reverse primer. PCR (Thermocycler; MJ Research PTC-200) was performed with 35 cycles by the following steps: denaturation at 94 °C for 30 s, annealing at 61 °C for 30 s, and extension at 72 °C for 1 min. After amplification the 256-bp PCR product was digested with FastDigest *Fok1* restriction endonuclease for 5 min. Digested products were analyzed on 2% agarose gel stained with ethidium bromide. The sizes of the bands were estimated using a 100-bp ladder. The genotyping was done on the basis of the presence or absence of the *Fok1* site as follows: FF=265 bp; Ff=265 bp, 169 bp, and 96 bp; and ff=169 bp and 96 bp lengths, respectively. The absence of a restriction site is represented by F while the presence of a restriction site is represented by f. 

The guanine (G) to thymidine (T) gene polymorphism in the Sp1-binding site in the first intron of the *Col1A1* gene was determined by a PCR-based method. The primers (MBI Fermentas, Lithuania) used for PCR to amplify *Col1A1* gene fragments were as follows; forward primer 5¢-TAACTTCTGGACTA TTTGCGGACTTTTTGG-3¢ and reverse primer 5¢-GTCCAGCCCTCATCCTGGCC-3¢ for the Sp1 restriction site DNA. Genotypes for *Col1A1* Sp1 polymorphisms were classified as G/G homozygotes (SS), G/T heterozygotes (Ss), and T/T homozygotes (ss).

### Statistical Analysis

In statistical analysis, SPSS 23.0 and Number Cruncher Statistical System 2007 were used. Categorical variables were defined with frequency and percentage; continuous variables were given as mean deviation in parametric conditions and median (min., max.) in nonparametric conditions. In the analytical review of the data, the effects of *Fok1* and *Col1A1* gene polymorphisms and other characteristics of the patient on low BMD, osteoporosis, osteonecrosis, and categorical variables were investigated by chi-square analysis. In chi-square analysis, Fisher’s exact test, Pearson’s chi-square test, the continuity correction test, and the Fisher-Freeman-Halton test were applied according to expected values and group numbers. Statistical significance was accepted at p<0.05.

## Results

The general characteristics of the patients are shown in Table 1. The median age at diagnosis was 61.5 months (min. 11, max. 204 months). Five (10%) patients had to be treated with HSCT. The median age of the patients at the time of first DEXA was 96 months (min. 35, max. 196 months). The median and mean DEXA Z-scores were 0.145 and 0.63 g/cm^2^, respectively. Low BMD, osteoporosis, and osteonecrosis were present in 18 patients (36%) (Table 2). Lumbosacral vertebras, femur heads, knees, and sacroiliac joints were the most affected areas on MRI. Osteonecrosis was present in one patient at the bilateral sacroiliac joints, one patient at the L4-L5 area, and two patients at the head of the left and right femur, respectively. 

The sex distribution among patients with bone features was not statistically significant. Rates of osteonecrosis and total bone changes were significantly higher in patients aged ≥10 years (p=0.001, p=0.029, respectively) (Table 2).

When the bone metabolism markers were examined, hypocalcemia was observed in 7 patients, hypophosphatemia was observed in 14 patients, and alkaline phosphatase elevation was observed in 12 patients. No significant difference was found in terms of risk of developing low BMD, osteoporosis, osteonecrosis, and total bone changes according to these markers. However, the risk of low BMD, osteonecrosis, and total bone changes was higher in those with vitamin D deficiency and this was statistically significant (p=0.003, p=0.004, p=0.001, respectively) (Table 3). There was no relationship between any of these bone changes and parameters that could change the duration of treatment, such as ALL risk groups, relapse status, and HSCT. Only one patient had osteonecrosis in our HSCT group.

Distribution of *Fok1* and *Col1A1* Sp1-binding site gene polymorphisms in both groups is shown in Table 4. *Fok1* polymorphism shows a significant difference between patients and the control group. In terms of the mentioned bone changes, *Col1A1* gene and *Fok1* Sp1-binding site gene polymorphisms did not show a significant correlation between BMD values and Z-scores. The distribution of *Fok1* and *Col1A1* Sp1-binding site gene polymorphisms according to low BMD, osteoporosis, and osteonecrosis is shown in Table 5. Only gene polymorphism in the Sp1-binding site of *Col1A1* showed a significant association in patients with osteonecrosis (p=0.045).

On follow-up, 2 of the 18 patients needed surgical operation; others received calcium and vitamin D supplements with or without bisphosphonate replacement therapy for better quality of life. All of the patients with low BMD and osteoporosis exhibited an increase in BMD values and Z-scores in the long-term follow-up period.

## Discussion

In children who had completed therapy for ALL, the prevalence of BMD abnormalities was reported to be as high as 93% and the incidence for asymptomatic osteonecrosis was found to vary between 15% and 38% among survivors [27,28,29]. Recently, Vitanza et al. [27] demonstrated that 46.6% of children exhibited osteoporosis in at least one anatomic site at some time during the first 6 years after chemotherapy. In our study, low BMD (32%), osteoporosis (24%), and osteonecrosis (16%) were present in 18 out of 50 patients (36%) who were followed for more than 7 years for these features. Lumbosacral vertebras, femur heads, knees, and sacroiliac joints were the most affected areas on MRI, as identified in the literature [28,29,30]. Prior studies reported multiple clinical risk factors for the development of osteonecrosis, including female sex, older age, administration of 3 weeks of continuous rather than alternate week dexamethasone during delayed intensiﬁcation, and intensive therapy [9,10,31,32,33]. We found no relationship between these bone changes and specific risk factors such as sex distribution, duration of treatment, ALL risk groups, and relapse status. As reported in the literature, the risk of osteonecrosis and other bone complications was significantly higher in patients aged ≥10 years in our study. Age remains the strongest and most consistently identiﬁed factor, with patients 10 to 20 years old at greatest risk [5,6,7,8,9,10,27,33]. In a retrospective report on the ALL-BFM-95 trial, the osteonecrosis incidence was reported to be 8.9% in patients aged ≥10 years and even higher in those ≥15 years (16.7%) [9]. The results also did not show female sex as a significant risk factor for developing osteonecrosis and higher incidences were found to be accompanied by higher total steroid doses. For the first time, Krull et al. [30] recently demonstrated that asymptomatic osteonecrosis develops independently of radiological leukemic infiltration of bone in adolescents with ALL. 

Different studies have reported a list of genes effective on osteoporosis, such as *VDR*, *Col1A1*, estrogen receptor alpha, interleukin-6, and LDL receptor-related protein 5 [16,17]. The relationships between *Col1A1* Sp1 polymorphism and BMD were investigated among various populations. Previous studies have shown associations between *Col1A1* Sp1 polymorphisms and low BMD, osteoporosis, and increased fracture risk [20,21,22,23,34,35], while some have not reached statistical significance [36,37]. In healthy prepubertal children, there have only been a small number of studies examining possible effects of *Col1A1* gene polymorphisms on BMD [38,39,40]. In our study, only gene polymorphism in the Sp1-binding site of *Col1A1* showed a significant association in ALL patients with osteonecrosis, but not for other bone abnormalities. Since the main component of bone matrix is collagen, this may be an important finding that has to be assessed in a larger group of ALL survivors. 

*VDR* *Fok1* locus polymorphism is considered to be a potential regulator of bone and calcium metabolism. Studies on *VDR* gene polymorphisms of the *Fok1* locus and its associations with bone mass in children have shown varied results [17,18,41,42,43]. Of note, *Fok1* locus polymorphism was found to be significantly different between patients and the control group in our study. This may have resulted from the relatively limited number of subjects in both groups. We found no significant association between *Fok1* genotypes and low BMD, osteoporosis, or osteonecrosis in children with ALL. In our study, the risk of low BMD, osteonecrosis, and total bone changes was higher in patients with vitamin D deficiency. Potential risk factors for decreased vitamin D in survivors of childhood cancer include poor diet, more time spent indoors, less physical activity, and administration of chemotherapy, steroids, and radiation therapy [44]. The prevalence of decreased vitamin D in children with cancer is high (29%-35%) but quite similar to what has been demonstrated in the general population [44,45]. A randomized double-blind study showed that nutritional counseling and vitamin D and calcium supplementation for 2 years offered no benefit for improving BMD among adolescent and young adult survivors of ALL [46]. Recently, a similar result was reported from a Turkish study group [47]. Depending on these findings, alternative and more aggressive strategies are needed to prevent these patients from experiencing bone complications.

## Conclusion

The development of therapy-induced bone mineral loss and osteonecrosis in children with ALL is frequent and the risk is higher especially in children aged ≥10 years and with vitamin D deficiency. The association between *Col1A1* Sp1-binding site gene polymorphisms and osteonecrosis has to be assessed in a larger group of ALL survivors. Studies investigating the possible underlying genetic susceptibilities to certain complications are important not only for better management of complications but also for development of new individual patient-specific treatment modalities.

## Figures and Tables

**Table 1:**
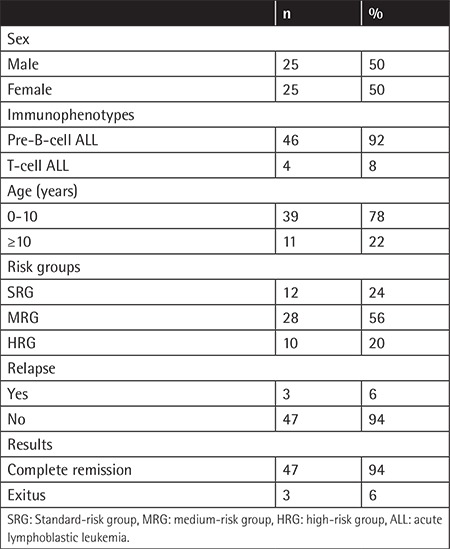
Demographic and clinical data of patients.

**Table 2:**
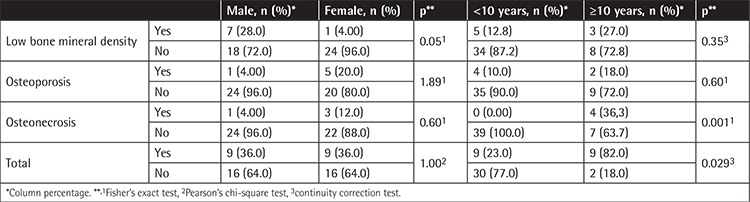
Distribution of bone abnormalities according to age and sex.

**Table 3:**
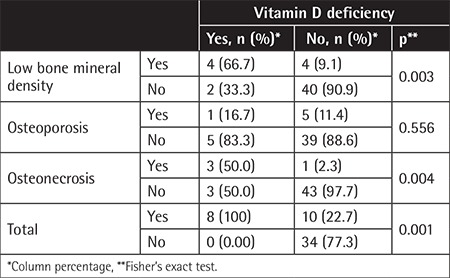
Correlation between vitamin D deficiency and bone changes.

**Table 4:**
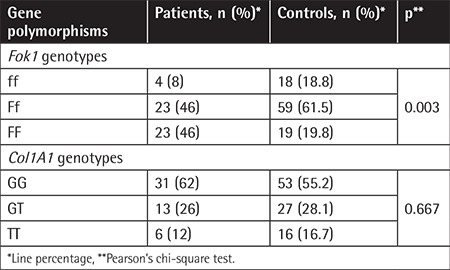
Distribution of *Fok1* polymorphism and *Col1A1* polymorphism in patients and control group.

**Table 5:**
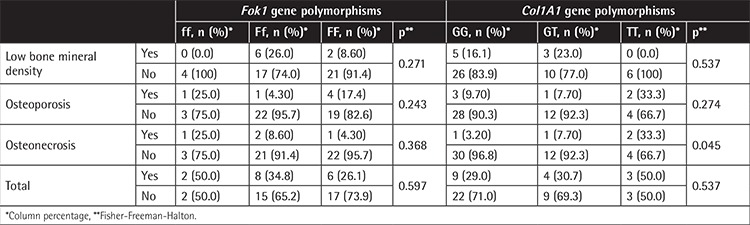
*Fok1* and *Col1A1* genotypes in children with bone abnormalities.
